# The influence of flow model selection on finite element model parameter estimation using Bayesian inference

**DOI:** 10.1121/10.0004260

**Published:** 2021-04-15

**Authors:** Paul J. Hadwin, Byron D. Erath, Sean D. Peterson

**Affiliations:** 1 Department of Mechanical and Mechatronics Engineering, University of Waterloo, Waterloo, Ontario N2L 3G1, Canada; 2 Department of Mechanical and Aeronautical Engineering, Clarkson University, Potsdam, New York 13699, USA pjhadwin@uwaterloo.ca, berath@clarkson.edu, peterson@uwaterloo.ca

## Abstract

Recently, Bayesian estimation coupled with finite element modeling has been demonstrated as a viable tool for estimating vocal fold material properties from kinematic information obtained via high-speed video recordings. In this article, the sensitivity of the parameter estimations to the employed fluid model is explored by considering Bernoulli and one-dimensional viscous fluid flow models. Simulation results indicate that prescribing an *ad hoc* separation location for the Bernoulli flow model can lead to large estimate biases, whereas including the separation location as an estimated parameter leads to results comparable to that of the viscous fluid flow model.

## 1. Introduction

The past decade has seen significant advances in the development of subject-specific vocal fold (VF) models.^1–8^ The development of subject-specific lumped element models was originally demonstrated by Dollinger *et al.*^1^ using the Nelder-Mead method to determine model parameters that best reproduced specifically chosen Fourier coefficients obtained from measured VF trajectory waveforms. Subject-specific models have been subsequently employed, for example, for speaker classification,^2^ contact force estimation,^8^ and subglottal pressure estimation.^3^

A wide variety of tools have been employed to generate subject-specific models, ranging from traditional least squares optimization approaches^1,2^ to machine learning-based methods.^4,5^ A particularly promising method employs the Bayesian framework to determine model parameters;^3,6,9^ this statistical method presents estimated parameters as probability density functions, thus providing additional information regarding estimate uncertainty.^10^ Bayesian estimation has been employed to estimate time-varying VF model parameters from synthetic VF kinematic data,^6,9^ subglottal pressure and muscle activation parameters from clinical data,^3^ and recently finite element material properties from high-speed video observations of vibrating silicone VF models.^7^

The success of such an estimation procedure is predicated on a variety of factors, including the quality of the observation data,^9,11^ the ability of the selected model(s) to capture the relevant physics, and the degrees of freedom of the estimation (i.e., the number of parameters to be estimated).^10,12^ Whereas prior work has employed a range of structural VF models of varying complexity,^1,2,5–7^ the driving fluid flow model has been exclusively confined to one-dimensional Bernoulli flow with an *ad hoc* fixed flow separation condition.^13,14^ Potential improvements to parameter estimation and uncertainty through appropriate choice of fluid model bears consideration.

In this article, we explore the influence of fluid flow model selection on the accuracy and uncertainty of two-dimensional finite element model parameter estimates based upon observations of silicone VF model kinematics for a small range of subglottal pressures. Specifically, we compare two flow models: the aforementioned one-dimensional Bernoulli flow model and a one-dimensional viscous flow model.^15,16^ Two permutations are considered for the Bernoulli flow model: (i) the flow separation location set *a priori* and (ii) the separation location included in the set of estimated parameters, and thus not known *a priori*. The viscous flow model is based upon a model of flow through collapsible tubes^17^ and naturally incorporates flow losses and separation. The model has been previously calibrated using three-dimensional fluid-structure interaction VF simulations and exhibited good agreement in terms of intra-glottal pressure distributions.^16^ Herein, higher fidelity computational fluid dynamics models are eschewed because the estimation procedure requires the solution of thousands of models, which would have an extremely high computational cost.

## 2. Methods

The observation data, finite element model, and Bayesian estimation procedure employed herein are identical to that reported by Hadwin *et al.*,^7^ except as noted in Sec. 2.2 below. This section contains a brief overview of the experimental setup and modelling methodologies; for a more detailed description, refer to Hadwin *et al..*^7^

### 2.1 Experimental setup and data collection

**Fig. 1.**
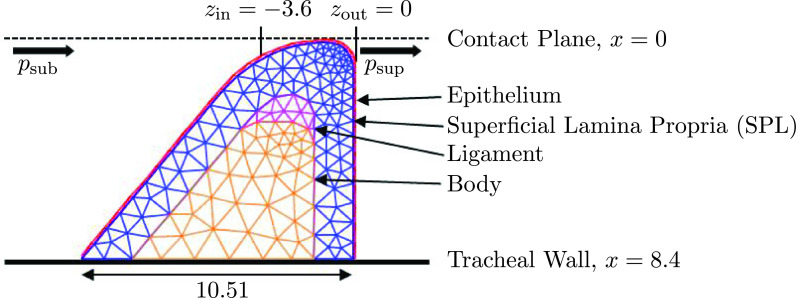
Schematic of the configuration for the numerical model of the silicone vocal folds with dimensions shown in mm.

The kinematics of a silicone VF analogue in a hemi-laryngeal configuration were recorded via high speed video using an 8-bit IDT MotionPro X3 PLUS camera equipped with an Elicar V-HQ Macro 90 mm lens for subglottal pressures of $p_{\text{sub}}=0.910$, 1.001, 1.092, and 1.183 kPa. The glottal area waveform was extracted using the standard segmentation approach.^18^ The VF analogues had a modified M5 geometry^19^ comprising four cross-sectional layers: the body, superficial lamina propria (SLP), ligament, and epithelium, with elastic moduli $E_{b}=11.8\;\text{kPa},\;E_{s}=0.6\;\text{kPa},\;E_{l}=2.0\;\text{kPa}$, and $E_{e}=45.0\;\text{kPa}$, respectively. These values were computed via uniaxial elongation test on cylinders made with the same silicone mixture ratios as was used for the different layers of the VF model.^20^ Strains ranging from 0% to ∼40% were applied and stress measured. Good linear behavior was observed between the stress and strain. The Young's modulus of elasticity was then computed as the slope from the line of best fit. The cross-sectional geometry of the silicone folds are shown schematically in Fig. 1.

The silicone VF was mounted in a custom hemi-laryngeal flow facility and driven from a compressed air supply that was down-regulated to the desired pressure. The air passed into an acoustically treated cylindrical plenum chamber, simulating a lung, which exhausted into a square tube, representing the trachea. The model trachea contained a flush-mounted pressure transducer upstream of the glottal entrance that measured $p_{\text{sub}}$. The silicone VF was mounted at the exit of the tracheal tube in a fixture that controlled the initial degree of medial compression. In all cases studied herein, the VF was mounted such that there was medial compression of m=0.4 mm, which specifies the distance the medial surface was compressed into the hemilaryngeal wall. This compression ensured full contact during phonatory collision. Anterior–posterior tensioning was applied in all cases by pulling on the string embedded in the models with a constant force of 0.3 N. No supraglottal tract was employed.

### 2.2 Numerical models

The experimental configuration is replicated in the numerical domain by coupling a finite element representation of the VFs with the fluids models to be tested. The subglottal tract of the experimental configuration was designed to replicate a human model,^21^ which has very low acoustic impedance and is thus not expected to influence the VF kinematics.^22^ Furthermore, since no supraglottal tract was employed in the experiments, acoustics are neglected herein. For reference, a Cartesian coordinate system is defined with the *x* axis pointing in the medial-lateral direction, the *y* axis in the posterior direction, and the *z*-axis in the superior-posterior direction, oriented to define a standard right-handed coordinate system.

#### 2.2.1 Solid model

The geometry of the silicone VF analogue is approximated using the triangulation seen in Fig. 1, which replicates the external geometry and internal cross-sectional layers of the manufactured silicone VF. For simplicity, the variation along the *y* axis is treated as uniform, thus yielding a 2D approximation. The domain generated by this geometry is denoted by Ω, with boundary Γ. The boundary is decomposed into two components: the surface that experiences the aerodynamic forces, $\Gamma_{f}$, and a fixed portion, $\Gamma_{\text{fixed}}$, which is fixed in time.

As discussed above, the material properties were found to be well approximated as linear and as the data has a limited range of subglottal pressures the silicone VF will be treated as a linear elastic material. A similar approach was taken by Alipour *et al.*,^23^ where the silicone VF is modeled as a linear isotropic viscoelastic body subject to a displacement field u(x,t) in the *xz* plane, where x∈Ω is a position vector in the deformed state and *t* is time. Employing an arbitrary variation $u^{\delta }$, the virtual work principle gives^24^

$$\int_{\Omega }{\left(u^{\delta }\right)}^{T}\rho_{b}(x)\overset{˙˙}{u}\;dV+\int_{\Omega }\sigma (u):\varepsilon (u^{\delta })\;dV=\int_{\Gamma_{f}}{\left(u^{\delta }\right)}^{T}f_{s}\;dS,$$ (1)

where *ρ_b_* is the material density, σ and ε are the Cauchy stress tensor and the strain tensor, respectively, and $f_{s}$ is the surface force, in this case arising solely from aerodynamic pressure acting on $\Gamma_{f}$. Under the assumption of linear elasticity, Hooke's law gives^24^

$$\sigma (u):\varepsilon (u^{\delta })=\tilde{\varepsilon }{\left(u^{\delta }\right)}^{T}C\tilde{\varepsilon }(u),$$ (2)

where $\tilde{\varepsilon }(u)={\left[\partial u_{x}/\partial x,\;\partial u_{z}/\partial z,\;\partial u_{z}/\partial x+\partial u_{x}/\partial z\right]}^{T}$ with subscripts *x* and *z* indicating vector components in the corresponding directions. The positive definite matrix **C** is given by

$$C(E,\nu )=\mu (E,\nu )\left(\begin{array}{ccc} \frac{2(1-\nu )}{1-2\nu } & \frac{2\nu }{\left(1-2\nu \right)} & 0\\ \frac{2\nu }{\left(1-2\nu \right)} & \frac{2(1-\nu )}{1-2\nu } & 0\\ 0 & 0 & 1 \end{array}\right),$$ (3)

where μ(E,ν)=E/(2(1+ν)) is the shear modulus, with *E* and *ν* being Young's modulus and Poisson's ratio, respectively. Following Alipour *et al.*,^23^ viscous damping is incorporated via a Kelvin-Voigt framework, wherein μ(E,ν) is replaced with μ+ηd/dt, where *η* is viscosity.

The finite element approximation of the displacement field is given by u(x,t)≈H(x)θ(t), where H(x) is a matrix of piecewise linear basis functions, and θ(t) is a coefficient vector that defines the temporal evolution. Substituting this into Eq. (1) gives

$$M\overset{˙˙}{\theta }+D\dot{\theta }+K\theta =F,$$ (4)

where M, D, and **K** are the mass, damping, and stiffness matrices, respectively, and **F** is the nodal force vector. Additionally, collisions are handled by enforcing no crossing of the contact plane, seen in Fig. 1. This means when a node in the model crosses the contact plane the *x*-coordinate of that node is forced back to the contact plane.

Finally, an initial medial compression of *m* mm is computed through modification of the initial state of each node. The modified initial state is calculated via Eq. (4) with time derivatives set to zero. This equation was solved iteratively by adjusting the *x*-coordinate of the nodes in steps of $10^{-4}\;\text{mm}$ then applying a force of $10^{-4}\;N$ to any node crossing the contact plan. Once the model has been shifted a total of *m* mm towards the midline the process is terminated.

See Secs. 3.2 and 4 from Hadwin *et al.*^7^ for a detailed description of the model.

#### 2.2.2 Fluid models

Two fluid models are considered herein, the traditional inviscid one-dimensional Bernoulli flow model with imposed flow separation,^13^ and a one-dimensional viscous flow model.^16,17^ In both cases the fluid loading is modeled as a normal surface force acting on $\Gamma_{f}$. For the Bernoulli flow model the intraglottal pressure distribution is given by

$$p(s,t)=\{\begin{array}{cc} p_{\text{sub}}-(p_{\text{sub}}-p_{\text{sup}}){\left(\frac{A_{\text{sep}}(t)}{A(s,t)}\right)}^{2}, & A(s,t)<A_{\text{sep}}(t),\\ p_{\text{sup}}, & A(s,t)\geq A_{\text{sep}}(t), \end{array}$$ (5)

where $s\in \Gamma_{f}$ represents the position along the surface where the aerodynamic forces are applied, $p_{\text{sub}}$ and $p_{\text{sup}}$ are the subglottal and supraglottal pressures, respectively, *A*(*s*, *t*) is the glottal area at location $s\in \Gamma_{f}$ at time *t*, and $A_{\text{sep}}$ is the glottal area at the location of flow separation.^13,25^ Typically the flow separation location is set at some point downstream of the minimal glottal area such that $A_{\text{sep}}(t)=r_{\text{sep}}A_{\text{min}}(t)$, where $r_{\text{sep}}\geq 1$ is a constant value and $A_{\text{min}}(t)={\text{min}}_{s\in \Gamma_{f}}A(s,t)$. This model is computationally efficient and has been shown capable of reproducing VF motion similar to that generated by solving the full Navier-Stokes equations.^26^ However, it has also been shown that quantities such as peak flow rate can be poorly predicted^26^ due to neglecting viscous losses and accurately modeling flow separation.^16^

The viscous flow model adapts a one-dimensional model of flow through collapsible tubes introduced by Cancelli and Pedley^17^ to approximate separation effects and viscous losses in the glottis.^15,16,27^ The viscous flow model is described by a pair of coupled partial differential equations^15,16^

$$\frac{\partial A}{\partial t}+\frac{\partial wA}{\partial z}=0,$$ (6)

$$\rho_{f}\frac{\partial w}{\partial t}+\rho_{f}w\frac{\partial w}{\partial z}=-\frac{\partial p}{\partial z}+\tau \frac{S}{A},$$ (7)

where $\rho_{f}=1.14\;{\text{kg}/m}^{3}$, *w*, and *p* are, respectively, the density, velocity, and pressure of the fluid (modeled as air at 1 atm and 36.5 °C), *A* is the effective area of the cross section, *S* is the perimeter of the cross-sectional area, and $\tau =\tau_{\text{fric}}+\tau_{\chi }$ is the total shear stress, which includes contributions from viscous ( $\tau_{\text{fric}}$) and flow separation losses ( $\tau_{\chi })$. The effective cross-sectional area is computed as $A(z)=\alpha (z)A_{0}(z)$, where $A_{0}(z)$ is the actual cross-sectional area and α(z) is a correction coefficient accounting for the *vena contracta* of the glottal jet. The resulting boundary value problem has boundary conditions $p_{\text{sub}}$ and $p_{\text{sup}}$ at the inlet and outlet, respectively. This boundary value problem is solved using typical shooting methods with the velocity having a zero derivative boundary condition upstream.^15,16^

The model behavior depends on the form of the shear stress, *τ*, and correction coefficient, α(z). Cancelli and Pedley^17^ modeled the loss of kinetic energy as

$$\tau_{\chi }=\frac{A}{s}\left(1-\chi \right)\rho_{f}w\frac{\partial w}{\partial z},$$ (8)

where 0≤χ≤1 is a constant representing pressure recovery. Specifically, *χ* = 1 represents no separation loss and *χ* = 0 results in no recovery. It is typical to set *χ* = 1 before the minimum glottal area and $\chi =\chi_{\text{min}}=0.2$ after this location.^15,27^ The viscous stress is modeled as fully developed flow in a tube of constant cross section, yielding $\tau_{\text{fric}}=-2\mu \left(S/A\right)u$, where $\mu =1.9\times 10^{-5}\;\text{kg}/(m\;s)$ is the dynamic viscosity of the fluid. For the correction coefficient, α(z), a quadratic function has been employed:^15,16^

$$\alpha (z)=0.75+0.25\frac{{\left(z-z_{\text{out}}\right)}^{2}}{{\left(z_{\text{in}}-z_{\text{out}}\right)}^{2}},\;z_{\text{in}}\leq z\leq z_{\text{out}},$$ (9)

where the subscripts “in” and “out” denote the locations of the inlet and outlet of the glottal channel. In the present study, the glottal inlet and outlet are at $z_{\text{in}}=-3.6\;\text{mm}$ and $z_{\text{out}}=0\;\text{mm}$, respectively; see Fig. 1 for positions.

### 2.3 Bayesian inference

Bayesian parameter inference computes a joint probability distribution which represents the probability of all potential values of the parameters of interest, *ξ*. This density is computed via Bayes Eq. (10),

$$\pi (\xi |y)=\frac{\pi (y|\xi )\pi_{\text{pri}}(\xi )}{\pi (y)}\propto \pi (y|\xi )\pi_{\text{pri}}(\xi ).$$ (10)

Here, π(ξ|y) is the posterior probability density function, which contains all probabilistic information about *ξ* given observed measurements **y**. The density $\pi_{\text{pri}}(\xi )$ is the “prior” probability density, π(y|ξ) is the “likelihood,” and π(y) is the “evidence.” The prior contains known or expected statistical properties of the parameters based on all available information prior to observation capture (e.g., subglottal pressure falls within a known expected range). The likelihood quantifies the probability of an observed measurement occurring for given parameter values. Finally, the evidence is a normalization constant ensuring that the law of total probability is satisfied.

In the present work, importance sampling is used due to the computational complexity of the model.^10^ Such approaches have been successfully used previously for the study of voice.^6,7,28,29^ Importance sampling is built on the principle that certain values of the input parameters are more important than others. So, a greater weight is allocated to those regions in the parameter space that exhibit a better statistical fit to the measurements. The weighting of the parameter space is determined by the value of the likelihood when random values of the model parameters are sampled from the prior, i.e., based on a measure of the goodness of fit. The samples from the prior are then re-sampled according to the weights and sample mean and standard deviations can be computed to generate estimates of the parameter values and uncertainties, respectively.

### 2.4 Estimates

The glottal area waveforms extracted from the high speed video recordings of the silicone VFs at each experimental subglottal pressure were used as the data for purposes of fitting the model. As the proposed model of the vocal folds is two-dimensional only a glottal width can be simulated. To convert the extracted glottal areas into a glottal width, the extracted area was divided by the peak length of the glottal opening ( ∼14 mm in all cases) giving a length averaged glottal width. This length averaged glottal width was then used to generate estimates of subglottal pressure $p_{\text{sub}}$, material density *ρ_b_*, damping coefficient *η*, initial medial compression *m*, and the elastic moduli for the body ( $E_{b}$), SLP ( $E_{s}$), and ligament ( $E_{l}$). Density and damping coefficient were treated as uniform across the VF layers. All simulations used a time step of 0.01 ms. The estimates were computed using an ensemble size of 15 000 samples generated from the prior for each estimate.^7^ Poisson's ratio and supraglottal pressure were treated as fixed and known with values of ν=0.4995 and $p_{\text{sup}}=0\;\text{kPa}$, respectively. Furthermore, stability issues arose when attempting to estimate epithelial stiffness $E_{e}$, and as such it was also treated as constant with its value set to that of the experimental VFs.

Eight permutations of the Bernoulli fluid model were considered. The first seven permutations employed a prescribed flow separation location with $r_{\text{sep}}$ ranging from 1.0 to 1.6 in increments of 0.1. The final Bernoulli model permutation included $r_{\text{sep}}$ as an estimated parameter. Since there is no *ad hoc* prescription of flow separation location in the viscous flow model only a single permutation of that model was considered.

The importance sampling algorithm allocates weights to the samples according to the likelihood density. This density is determined by the error model used for the data. In this work, uncertainties associated with the experimental measurements are treated as unbiased additive and normally distributed with a standard deviation of $1\;{\text{mm}}^{2}$. This corresponds to a relatively large noise level of approximately 6%; such a large noise level was chosen to “whiten” the likelihood to account for model errors that are present.^10^

## 3. Results and discussion

**Fig. 2.**
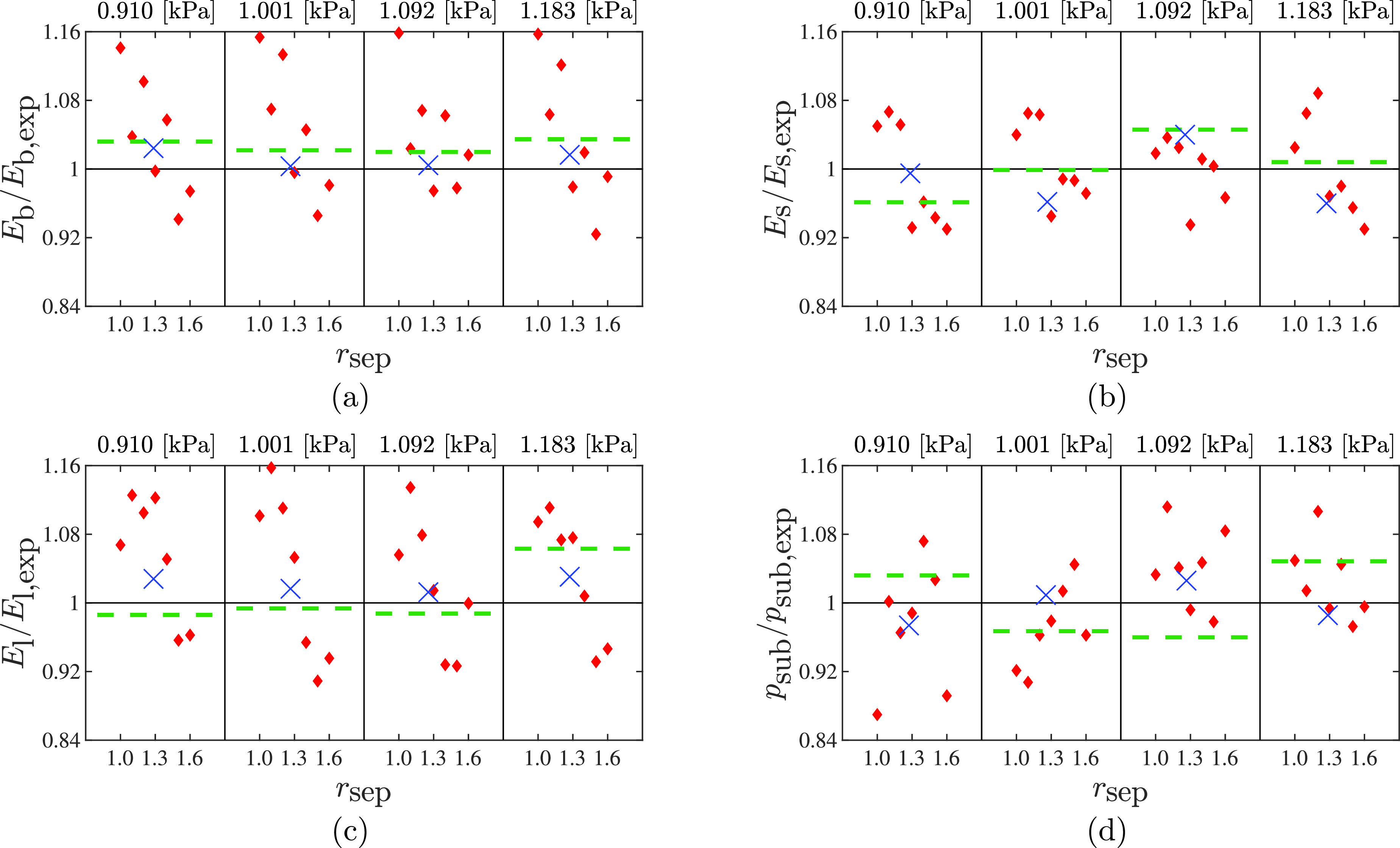
The estimated values normalized by the experimental value for (a) $E_{b}$ with experimental value of $E_{b,\;\text{exp}\;}=11.8\;\text{kPa}$, (b) $E_{s}$ with experimental value of $E_{s,\;\text{exp}\;}=0.6\;\text{kPa}$, (c) $E_{l}$ with experimental value of $E_{l,\;\text{exp}\;}=2\;\text{kPa}$, and (d) $p_{\text{sub}}$. The title above each column indicates the experimental subglottal pressure. The solid line indicates a normalized value of one. Diamond markers indicate estimated values for the Bernoulli flow model with prescribed $r_{\text{sep}}$, with values given on the horizontal axis; cross-markers represent estimated values for the Bernoulli model with $r_{\text{sep}}$ estimated, the estimated value of $r_{\text{sep}}$ is marked on the horizontal axis; and the dashed lines represent estimates using the viscous flow model.

Estimates of the elastic moduli and subglottal pressure are presented in Fig. 2 for each experimental data set and all considered fluids models. The estimated values are normalized by their respective experimental values, which are given in the caption for reference. The full set of results, including initial medial compression *m*, average material density *ρ_b_*, and damping coefficient *η*, are included as supplemental material.^30^

First considering the estimates using the Bernoulli flow models with prescribed separation location (diamond symbols) highlights the sensitivity of the estimation process to the selection of the separation ratio, $r_{\text{sep}}$. Specifically, the results indicate that use of an incorrect fixed value for the separation ratio can lead to large biases in estimated parameter values. The elastic moduli were all overestimated for $r_{\text{sep}}\leq 1.2$ and underestimated for $r_{\text{sep}}\geq 1.4,$. Values in the range of 1.2 to 1.4 provided the most accurate estimates. There is, however, significant variability from case to case in the estimation accuracy across the three elastic moduli. The estimates for $E_{b}$ have a bias ranging from 1% to 15% whereas the bias in $E_{s}$ ranges from 1% to 8%.

Including the separation ratio as a parameter (cross) yields an estimated value of $r_{\text{sep}}\approx 1.27\pm 0.1,$ for all cases. This is in good agreement with the observations from the fixed $r_{\text{sep}}$ estimates in that the 95% confidence intervals fall largely between $r_{\text{sep}}=1.2$ and 1.4, which was the range of best estimation accuracy for those estimates. Additionally, the resulting elastic moduli estimates have good agreement with the experimental values, with a maximum bias of around 4%. As noted above, the estimates for $E_{s}$ are in general the most accurate, however it is the parameter with the greatest bias when $r_{\text{sep}}$ is estimated. Because $r_{\text{sep}}$ determines the proportion of $\Gamma_{f}$ over which the fluid forces act (beyond the separation location the applied pressure is fixed at $p_{\text{sup}}$), this indicates that changes in $r_{\text{sep}}$ can be slightly offset via a bias in $E_{s}$.

The viscous flow model (dashed line) produces estimates similar to those of the Bernoulli model when $r_{\text{sep}}$ is estimated, having a maximum bias of approximately 5%. The correction coefficient and shear stress functions are established via comparison with fully three-dimensional fluid-structure interaction simulations. Their approximate nature likely introduces an implicit error into the viscous flow model that propagates into the estimated values leading to the observed biases. Additionally, the viscous flow model was calibrated using a three-dimensional finite element model whereas the present simulations employ a two-dimensional model. It may be the case that if one generated new polynomial models for the correction coefficient and the shear stress specific to a two-dimensional finite element model this bias would be reduced and the viscous flow model may out-perform the Bernoulli model.

Trends in subglottal pressure for the Bernoulli models with fixed separation are less consistent across the four data sets with increasing $r_{\text{sep}}$, though the best estimates still occur in the range of $1.2\leq r_{\text{sep}}\leq 1.4$. The spread in the bias decreases as the experimental subglottal pressure increases. This is similar to the result found in Hadwin *et al.*,^7^ wherein the higher subglottal pressures had lower uncertainty, likely due to a higher sensitivity of the model to subglottal pressure as it rises.

**Fig. 3.**
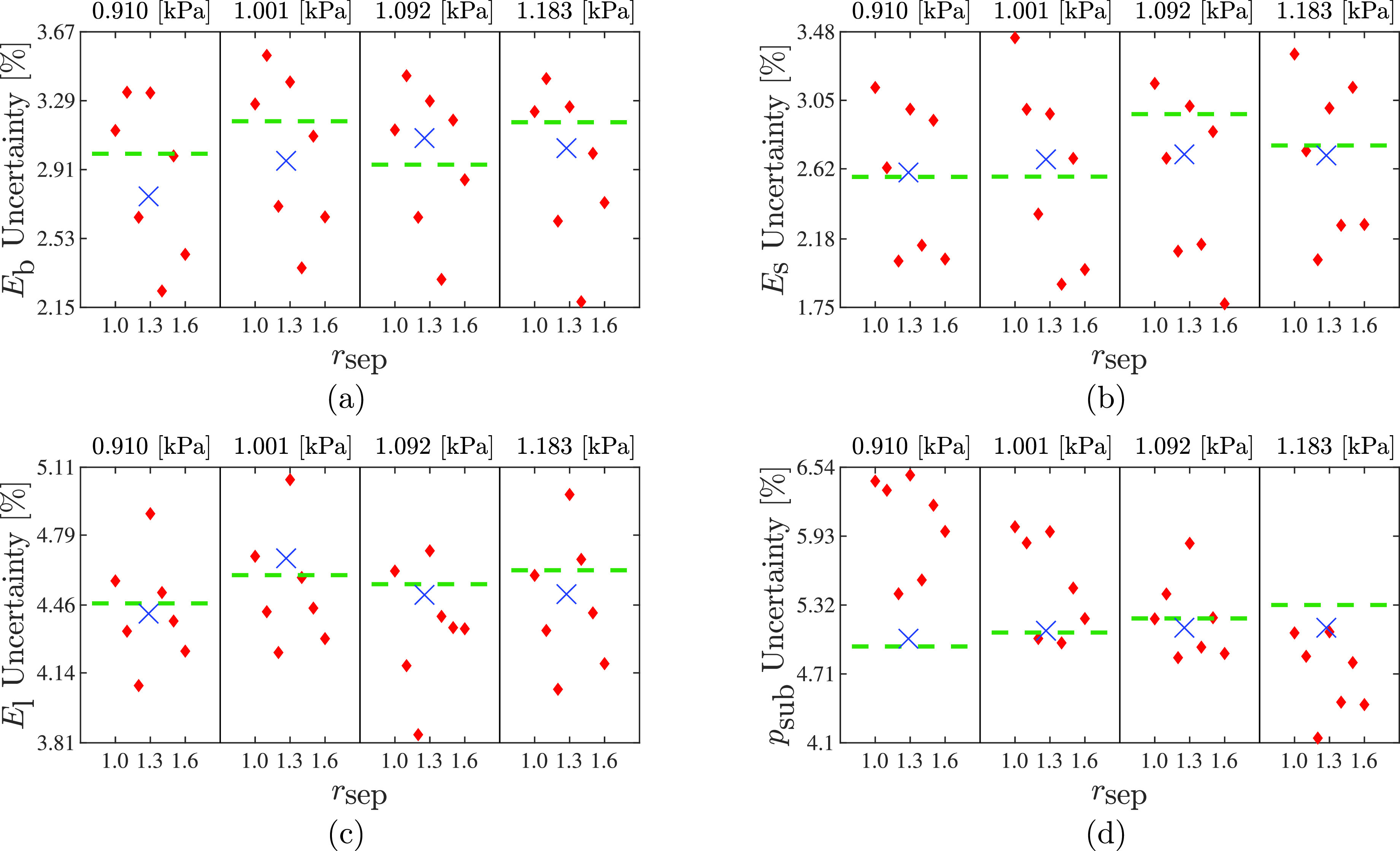
Estimated uncertainty, defined as the estimated standard deviation as a percentage of the estimated value, for (a) $E_{b}$ with experimental value of $E_{b,\;\text{exp}\;}=11.8\;\text{kPa}$, (b) $E_{s}$ with experimental value of $E_{s,\;\text{exp}\;}=0.6\;\text{kPa}$, (c) $E_{l}$ with experimental value of $E_{l,\;\text{exp}\;}=2\;\text{kPa}$, and (d) $p_{\text{sub}}$. The title above each column indicates the experimental subglottal pressure. Diamond markers indicate estimated values for the Bernoulli flow model with prescribed $r_{\text{sep}}$, with values given on the horizontal axis; cross-markers represent estimated values for the Bernoulli model with $r_{\text{sep}}$ estimated, the estimated value of $r_{\text{sep}}$ is marked on the horizontal axis; and the dashed lines represent estimates using of the viscous flow model.

While estimate bias may exist, in general, the estimated uncertainty of each model is sufficiently broad that the experimental values fall within two standard deviations with 95% confidence. Figure 3 presents the estimated relative uncertainty computed as the estimated standard deviation divided by the estimated value for all cases shown in Fig. 2. Overall, the level of estimated uncertainties are relatively small, with a maximum of around 6.5%; similar to the estimates, uncertainty decreases as subglottal pressure increases.

When $r_{\text{sep}}$ is fixed the uncertainties in the elastic moduli tend to oscillate as $r_{\text{sep}}$ increases. This is likely due to the ensemble size used with the importance sampling. As was seen in our previous work, the uncertainties stabilize when approximately 30 000 samples are used.^7^ The ensemble size of 15 000 samples was employed herein as we previously found that this number of samples resulted in less than a 20% increase in the estimated uncertainties and had minimal impact on bias, yet reduced the computational time by 50%.

The estimated uncertainties from the Bernoulli model with estimated separation location and the viscous flow model exhibit considerably lower and more stable values across conditions. Using the Bernoulli model with estimated $r_{\text{sep}}$ or viscous flow model gives approximately the same level of uncertainty, with the two estimates often converging as subglottal pressure increases. This indicates that they are both more consistent models to use in comparison with fixing $r_{\text{sep}}$.

In aggregate, these results demonstrate that employing an *a priori* fixed value for $r_{\text{sep}}$ results in high levels of variability and no clear trend in terms of both bias and uncertainty in the estimates. While a particular choice of $r_{\text{sep}}$ may yield an accurate estimate or low uncertainty, there is no way of discerning in advance what value of $r_{\text{sep}}$ to use. Additionally, there is no guarantee that the selected value will be optimal for each new set of observations. These problems can be avoided through the use of a more complex fluids model (the viscous flow model) or by including the separation ratio as an estimated parameter with the Bernoulli flow model. These results indicate that, in terms of computing estimates with the approximate 2D FE model, if the bulk of the flow is modeled with sufficient accuracy then material property estimates will also be accurate. With the similarity in results between the Bernoulli model with estimated separation location and the viscous flow model, and given that the viscous flow model comes at a 20% increase in computational expense, this study suggests the inviscid model is superior. However, it is worth noting that the tested data set is not exhaustive, and the experimental values of the material properties treat the silicone VFs as purely elastic. It may well be that the estimates using the viscous flow model would be superior in cases of higher subglottal pressures when the non-linearities of the material play a larger role. Additionally, more work is needed to determine whether using a non-linear material model would improve parameter estimates and uncertainties. Generally speaking, a more accurate model typically leading to a reduction in uncertainties, but uncertainty is a second order effect and non-linearity typically increases overall uncertainty.^10^

Finally, we note that while the employed two-dimensional finite element model results in estimates that are in good agreement with the experimental values, it remains to be determined whether accuracy can be improved by using a three-dimensional fitting model. This would be of particular potential relevance for experimental observations with considerable anterior-posterior variability.

## 4. Conclusion

This paper explored the sensitivity of subject-specific model parameter estimates to the selected fluid flow model. Both the standard Bernoulli flow model with fix flow separation location and a one-dimensional viscous flow model were considered. Employing glottal area waveforms obtained from high-speed video recordings of silicone vocal fold vibrations for a limited range of subglottal pressures as observation data, material properties of a 2D linearly elastic finite element vocal fold model, as well as driving subglottal pressure, were estimated via Bayesian inference. Results demonstrated that the estimated parameters were in good agreement with the values derived from a uniaxial elongation test when using the viscous flow model, as well as the Bernoulli flow model where the separation location was included in the set of estimated parameters.

Further work is necessary to assess whether the observations of this work extend to a broader range of operating parameters. While this work treats the silicone VF model as a linear elastic material and a small range of subglottal pressures are considered, the results are promising. They suggest that the relatively lower computational cost and comparable accuracy of the Bernoulli flow model with estimated separation location may be sufficient to produce meaningful estimates of material properties. Increased model complexity, including consideration of a three-dimensional fitting model, may be needed for operating parameters outside of the range studied here, as other higher-order modes may become important. Such issues will be considered in future work.
